# Supplementary material for: Sensory-motor cortices shape functional connectivity dynamics in the human brain
Source: Nat Commun. 2021 Nov 4;12:6373. doi: 10.1038/s41467-021-26704-y (PMC8568904; doi:10.1038/s41467-021-26704-y)
Supplement: Supplementary file 1 — Supplementary Information [file 41467_2021_26704_MOESM1_ESM.pdf]

# SUPPLEMENTARY INFORMATION

## Sensory-Motor Cortices Shape Functional Connectivity Dynamics in the Human Brain

Xiaolu Kong[1,2,3], Ru Kong[1,2,3], Csaba Orban[1,2,3], Wang Peng[4], Shaoshi Zhang[1,2,3,5], Kevin Anderson[6], Avram Holmes[7,8], John D. Murray[8], Gustavo Deco[9,10], Martijn van den Heuvel[11], B.T. Thomas Yeo[1,2,3,5,12]

[1]Department of Electrical and Computer Engineering, National University of Singapore, Singapore; [2]Centre for Sleep & Cognition & Centre for Translational Magnetic Resonance Research, Yong Loo Lin School of Medicine, Singapore; [3]N.1 Institute for Health & Institute for Digital Medicine, National University of Singapore, Singapore; [4]Max Planck Institute for Human Cognitive and Brain Sciences; [5]Integrative Sciences and Engineering Programme (ISEP), National University of Singapore, Singapore, Singapore [6]Department of Psychology, Center for Brain Science, Harvard University, Cambridge, USA [7]Department of Psychology, Yale University, New Haven, USA [8]Department of Psychiatry, Yale University, New Haven, USA; [9]Center for Brain and Cognition, Department of Technology and Information, Universitat Pompeu Fabra, Barcelona, Spain; [10]Institució Catalana de la Recerca i Estudis Avançats, Universitat Barcelona, Barcelona, Spain; [11]Department of Complex Trait Genetics, Amsterdam University Medical Center, Amsterdam, The Netherlands; [12]Athinoula A. Martinos Center for Biomedical Imaging, Massachusetts General Hospital, Charlestown, USA

**Address correspondence to:**

B.T. Thomas Yeo

ECE, CSC, TMR, N.1, WISDM

National University of Singapore

Email: thomas.yeo@nus.edu.sg

Table S1. Model performance in the HCP training, validation, and test sets. The HCP training set was used to generate 5000 MFM parameter sets, which were then evaluated in the HCP validation set. The top 10 candidates from the validation set were then evaluated in the HCP test set. The table below shows the performance (mean ± std) of these top 10 candidates in the HCP training, validation and test sets. Higher FC correlations and lower FCD KS statistics indicate better fit. The last column showed the p values of two-sample two-tail t-tests between pMFM and other parameterizations or cost function in the test set. Bolded results indicate statistically significant results after false discovery rate (FDR) correction (q < 0.05). Our approach (pMFM) was statistically better than (or comparable to) all other parameterizations in terms of FC correlation and/or FCD KS statistic in the test set. Optimizing for only fit to static FC (last row in table) led to slightly better (but statistically significant) fit to static FC in the test set, but much worse (and statistically significant) fit to FCD in the test set. Source data are provided as a Source Data file.

| | FC Correlation Training | FC Correlation Validation | FC Correlation Test | FC p-value Test Set | FCD KS Statistics Training | FCD KS Statistics Validation | FCD KS Statistics Test | FCD p-value Test |
|---|---|---|---|---|---|---|---|---|
| pMFM | 0.66±0.03 | 0.67±0.03 | 0.66±0.03 | | 0.04±0.02 | 0.03±0.01 | 0.12±0.03 | |
| Gradient only | 0.63±0.06 | 0.63±0.06 | 0.63±0.06 | 0.188 | 0.15±0.15 | 0.20±0.18 | 0.31±0.26 | **0.031** |
| T1w/T2w only | 0.56±0.04 | 0.56±0.04 | 0.55±0.03 | **2.0e-5** | 0.07±0.05 | 0.09±0.05 | 0.15±0.06 | 0.123 |
| Constant w, I & σ | 0.58±0.03 | 0.55±0.04 | 0.55±0.05 | **2.4e-5** | 0.28±0.30 | 0.31±0.29 | 0.50±0.31 | **0.001** |
| Constant w | 0.63±0.03 | 0.64±0.03 | 0.62±0.03 | **0.0245** | 0.05±0.02 | 0.07±0.04 | 0.11±0.04 | 0.729 |
| Constant I | 0.58±0.06 | 0.59±0.07 | 0.58±0.06 | **0.0037** | 0.11±0.06 | 0.13±0.08 | 0.14±0.06 | 0.179 |
| Constant σ | 0.57±0.03 | 0.59±0.04 | 0.57±0.04 | **2.48e-5** | 0.09±0.03 | 0.08±0.03 | 0.24±0.21 | 0.088 |
| Non-parametric | 0.66±0.02 | 0.66±0.03 | 0.66±0.03 | 0.754 | 0.06±0.03 | 0.04±0.02 | 0.28±0.11 | **2.1e-4** |
| FC Cost only | 0.74±0.005 | 0.76±0.002 | 0.73±0.01 | **2.1e-6** | 0.87±0.005 | 0.87±0.005 | 0.88±0.01 | **7.1e-23** |

Table S2. Table of correlations between FCD-STD correlational spatial maps and two gene expression maps: PVALB-SST and first principal component of gene expression[1,2]. P values that survived the false discovery rate (q < 0.05) are bolded. Standard deviations reported in the table were obtained by bootstrapping. Source data are provided as a Source Data file.

| | **PVALB-SST** | **p-value (spin test)** | **p-value (random gene pair)** | **Gene PC1** | **p-value (spin test)** |
|---|---|---|---|---|---|
| **FCD-STD Empirical** | 0.433±0.010 | **0.026** | **0.027** | 0.405±0.011 | 0.058 |
| **FCD-STD Simulated** | 0.544±0.007 | **0.006** | **0.021** | 0.556±0.007 | **0.005** |
| ***w*** | -0.508 | **0.005** | 0.060 | -0.550 | **0.004** |
| ***I*** | 0.320 | **0.042** | 0.118 | 0.330 | **0.037** |
| ***σ*** | -0.479 | **0.005** | 0.067 | -0.514 | **0.004** |

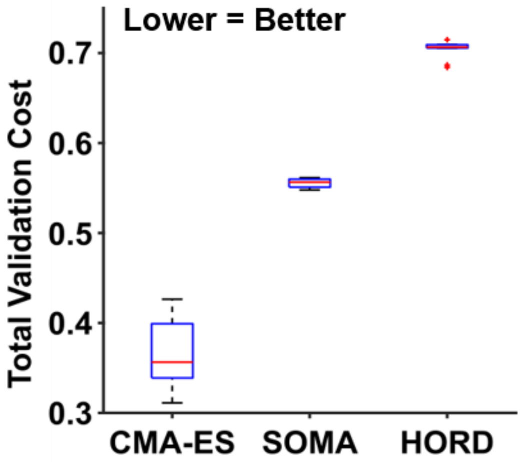


Figure S1. Comparison of three different algorithms: covariance matrix adaptation evolution strategy (CMA-ES[3]), self-organising migrating algorithm (SOMA[4]) and hyperparameter optimization using radial basis functions and dynamic coordinate search (HORD[5]) in the HCP validation set. Each algorithm was run on the training set generating 5000 candidate sets of model parameters. The 5000 candidate sets were evaluated in the validation set. The top 10 candidate sets from each algorithm (based on the validation set) are shown in this plot. Thus, CMA-ES performs the best among the three algorithms in the validation set. Box plots utilized default Matlab parameters, i.e., box shows median and inter-quartile range (IQR). Whiskers indicate 1.5 IQR. Red crosses represent outliers. Source data are provided as a Source Data file.

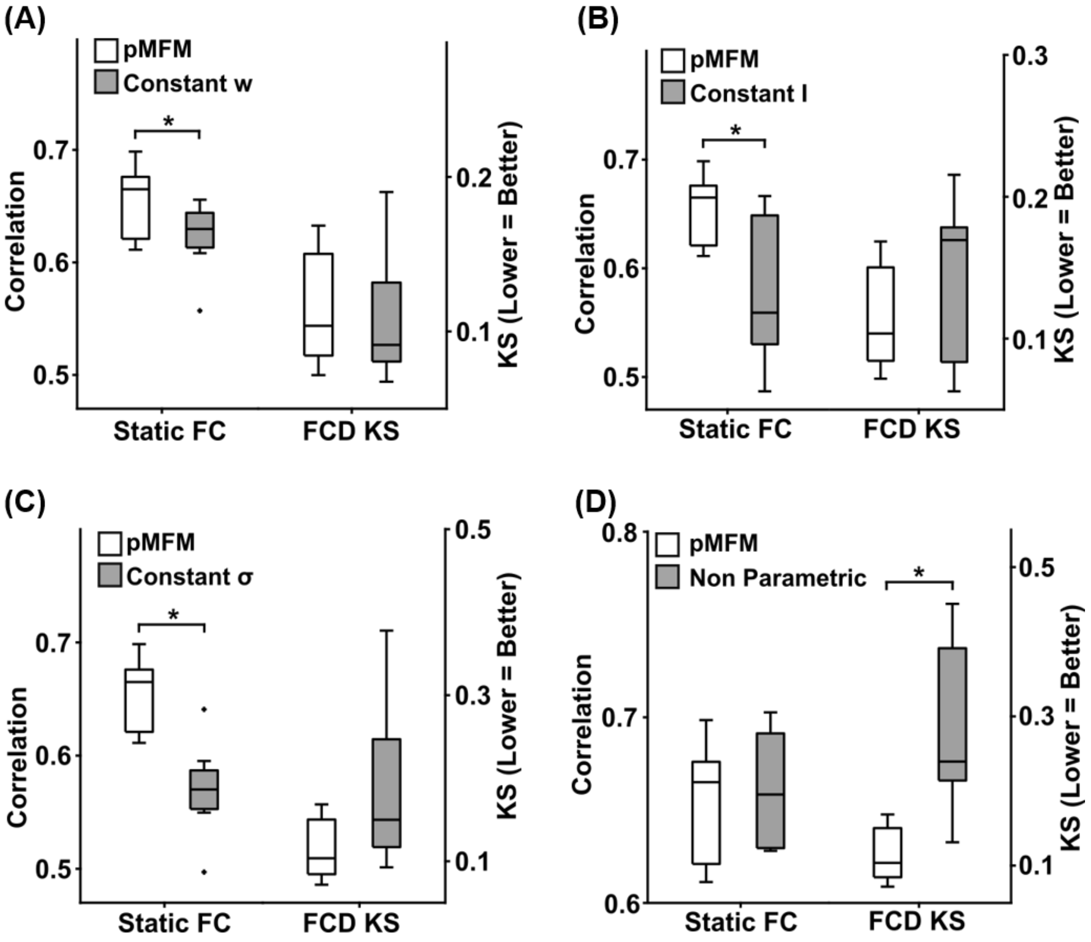


Figure S2. Comparison between the original pMFM (main text) and (A) constraining recurrent connection strength $w$ to be constant across ROIs, (B) constraining external input $I$ to be constant across ROIs, (C) constraining noise amplitude $\sigma$ to be the same across ROIs, and (D) allowing local circuit parameters to vary independent (i.e., not parameterized by anatomical and/or functional gradients). Across all panels, agreement between simulated and empirical static FC was measured using Pearson's correlation, while disagreement between simulated and empirical FCD was measured using KS distance. Across all analyses, top ten model parameter sets were selected from the validation set and applied to the test set. Each boxplot comprises 10 correlation values (left) or 10 KS statistic (right) based on the 10 best candidate sets from the validation set. The boxes show the inter-quartile range (IQR) and the median. Whiskers indicate 1.5 IQR. Black crosses represent outliers. * indicates statistical significance after multiple comparisons correction with false discovery rate (FDR) $q < 0.05$. Source data are provided as a Source Data file.

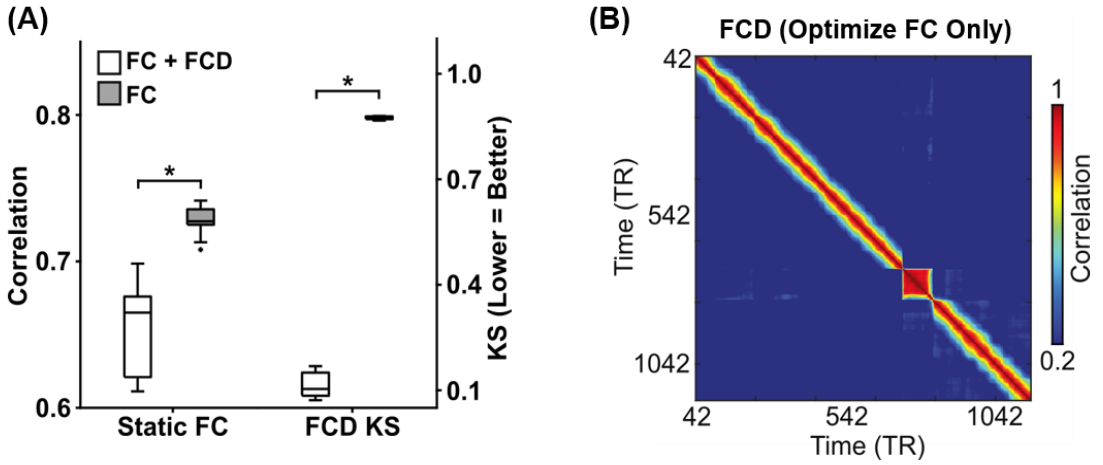


Figure S3. Comparison between the original pMFM (optimized using both static FC and FCD) and pMFM optimized using only static FC. (A) Agreement (Pearson's correlation r) between simulated and empirically observed static FC, as well as disagreement (KS distance) between simulated and empirically observed FCD. Each boxplot comprises 10 correlation values (left) or 10 KS statistic (right) based on the 10 best candidate sets from the validation set. The boxes show the inter-quartile range (IQR) and the median. Whiskers indicate 1.5 IQR. Black crosses represent outliers. * indicates statistical significance after multiple comparisons correction with false discovery rate (FDR) $q < 0.05$. (B) Simulated FCD from the pMFM optimized only using static FC. The simulated FCD was a lot less realistic than the original pMFM (Figure 2B). In terms of KS distance, there is a large improvement when optimizing both static FC and FCD (KS = 0.12 versus 0.88). However, when optimizing only static FC, the resulting simulated static FC was only slightly better than the original pMFM (r = 0.74 versus 0.66). This suggests that the goals of generating realistic static FC and FCD were not necessarily contradictory. We note that across all analyses, top ten model parameter sets were selected from the validation set and applied to the test set. The error bars correspond to standard error across the 10 parameter sets. Source data are provided as a Source Data file.

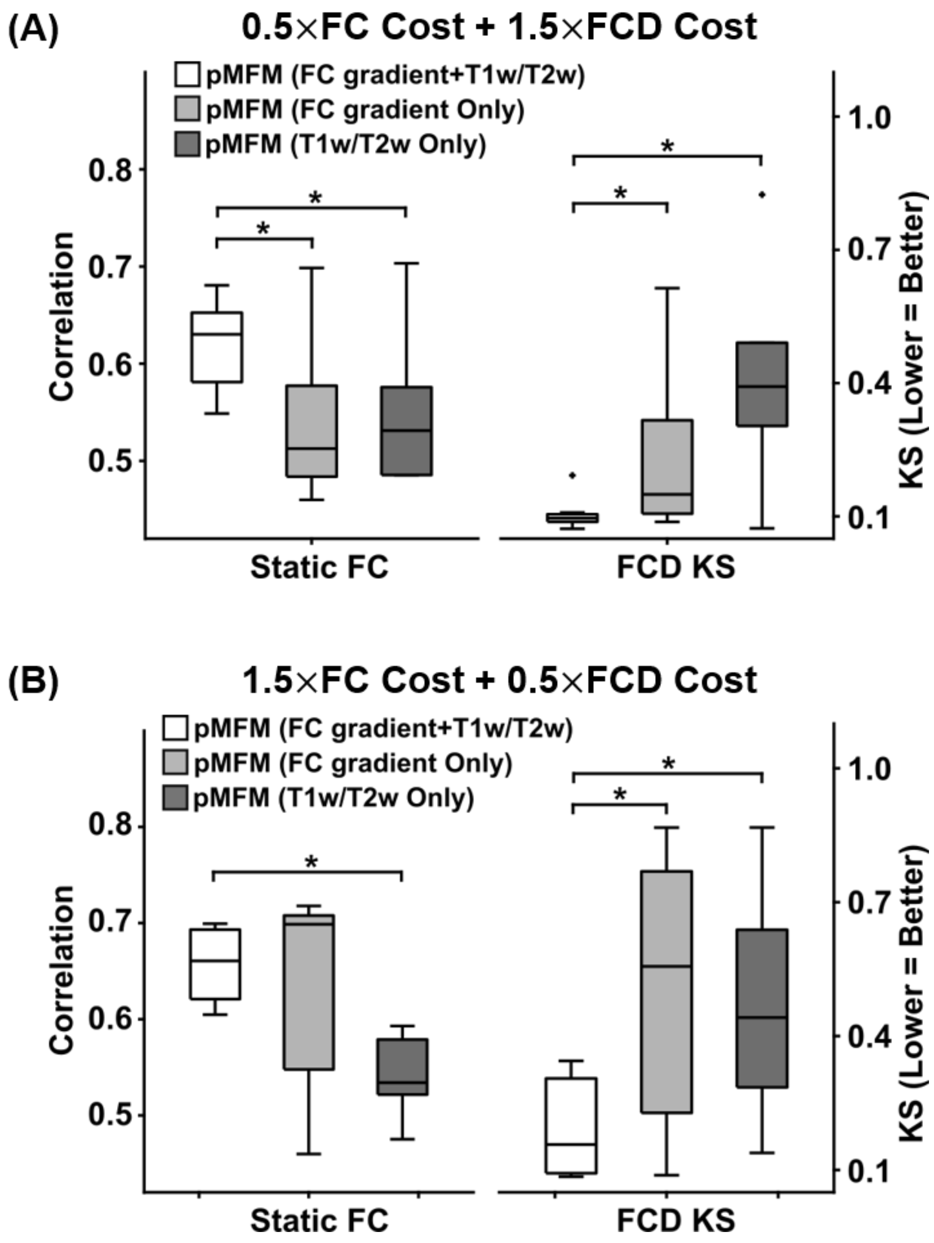


Figure S4. Combining both T1w/T2w map and FC gradient yielded better performance even when the relative weights of static FC cost and FCD cost in the cost function were altered. (A) Stronger weight on FCD cost. (B) Stronger weight on FC cost. Not surprisingly, increasing the weight of the FC cost (panel B) yielded better (greater) static FC correlation and worse (greater) FCD KS statistic in the test set. Each boxplot comprises 10 correlation values (left) or 10 KS statistic (right) based on the 10 best candidate sets from the validation set. The boxes show the inter-quartile range (IQR) and the median. Whiskers indicate 1.5 IQR. Black crosses represent outliers. * indicates statistical significance after multiple comparisons correction with false discovery rate (FDR) $q < 0.05$. Source data are provided as a Source Data file.

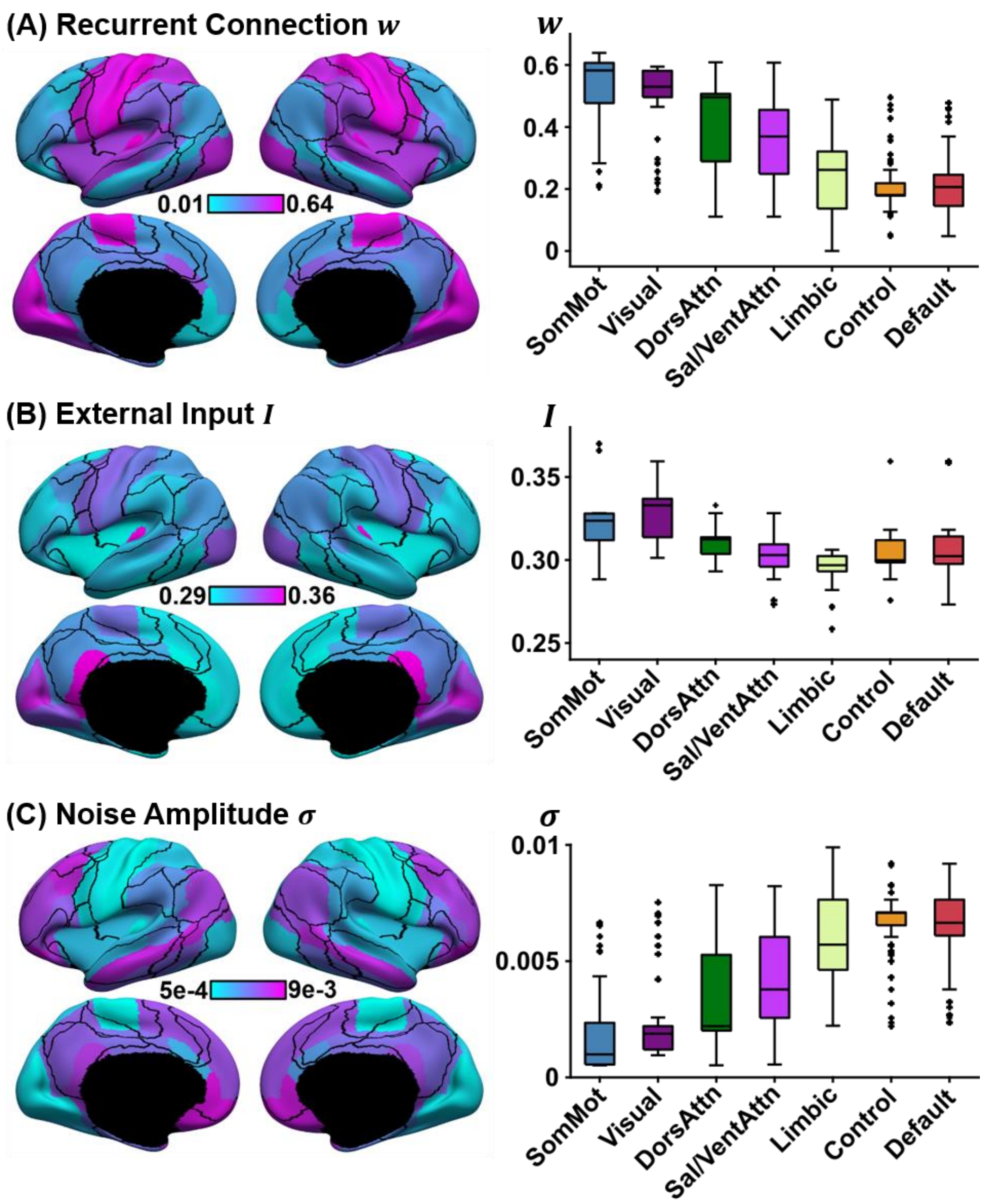


Figure S5. For one of the top ten parameter sets, recurrent connection strength exhibited the opposite direction from the remaining nine parameter sets. The layout of this figure is the same as Figure 4. (A) Strength of recurrent connection $w$ in 68 Desikan-Killiany cortical ROIs (left) and seven resting-state networks (right). (B) Strength of external input $I$ in 68 Desikan-Killiany cortical ROIs (left) and seven resting-state networks (right). (C) Strength of noise amplitude $\sigma$ in 68 Desikan-Killiany cortical ROIs (left) and seven resting-state networks (right). The boxplots comprised values obtained by "transferring" the parameter estimates from the 68 Desikan-Killiany parcels to all vertices (from the underlying cortical meshes) comprising each anatomical parcel. The vertex wise parameter values were then segregated based on seven resting-state networks. Therefore, there were 3203, 2478, 1523, 1520, 1067, 1438 and 2886 values comprising the boxplots for somatomotor, visual, dorsal attention, ventral attention, limbic, control and default networks

respectively. The boxes show the inter-quartile range (IQR) and the median. Whiskers indicate 1.5 IQR. Black crosses represent outliers. Noise amplitude increased from sensory-motor to association (limbic, control and default) networks. On the other hand, external input current and recurrent connection strength decreased from sensory-motor to association networks. Source data are provided as a Source Data file.

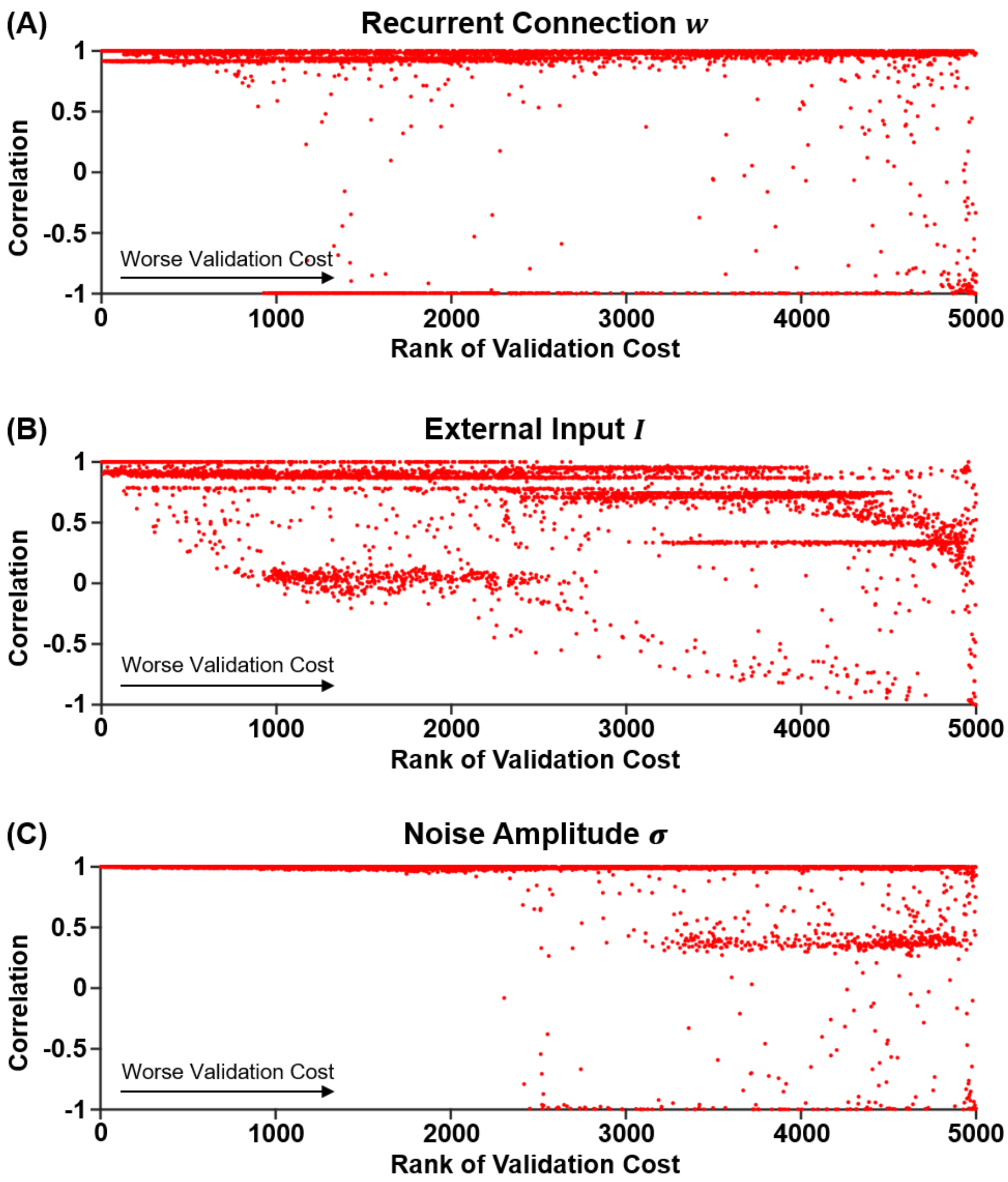


Figure S6. Correlations of local circuit parameter maps between the top parameter set from the validation set and the remaining 4999 candidate parameter sets. The 4999 candidate parameter sets were ordered based on their performance in the validation set. In general, parameter sets with good validation cost were strongly correlated with the top parameter estimate from the validation set. In the case of recurrent connection $w$, the correlations between the top 400 estimates of $w$ and the top estimate of $w$ were more than 0.9. In the case of external input $I$, the correlations between the top 60 estimates of $I$ and the top estimate of $I$ were more than 0.9. In the case of noise amplitude $\sigma$, the correlation between the top 2000 estimates of $\sigma$ and the top estimate of $\sigma$ were more than 0.9. In the case of external input $I$. Therefore, external input appeared to be less stable than recurrent connection strength and noise amplitude. Source data are provided as a Source Data file.

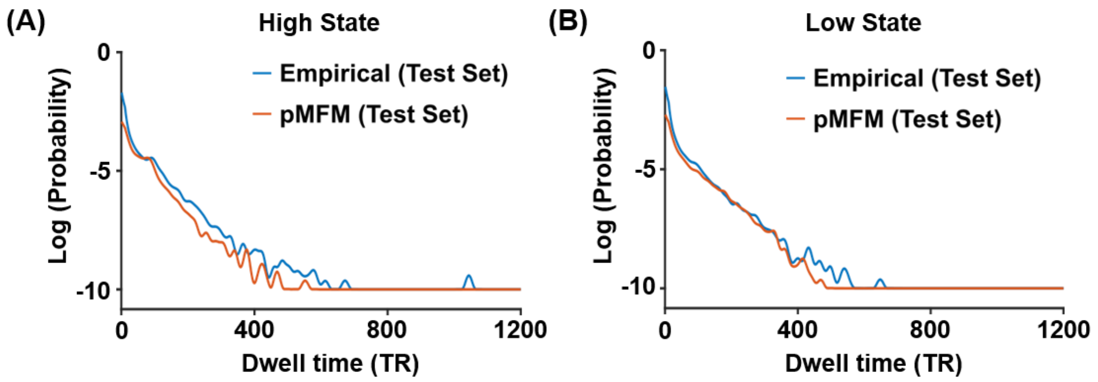


Figure S7. Distributions of dwell time for coherent (high FCD mean) and incoherent (low FCD mean) states in both empirical and pMFM-simulated results. The dwell time distributions of the two states were similar between empirical and simulated data. Furthermore, the two distributions appeared to follow an exponential distribution (as opposed to a gamma distribution), suggesting the presence of multi-stability rather than meta-stability[6]. Source data are provided as a Source Data file.

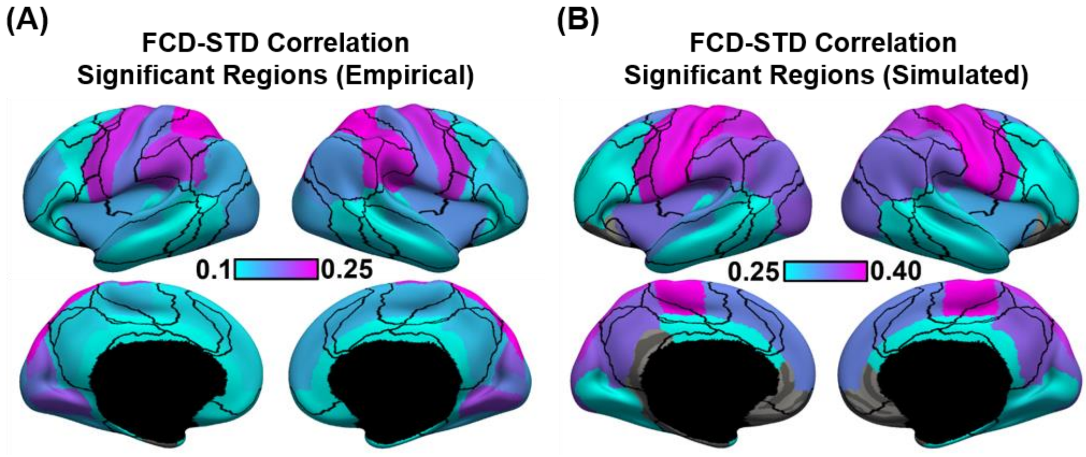


Figure S8. Sensory-motor regions drive sharp transitions in functional connectivity dynamics (FCD). (A) FCD-STD correlations obtained by correlating the first derivative of the FCD mean time course and the first derivative of the SW-STD time course of each cortical region. These correlations were performed for each HCP test participant and averaged across all runs and participants. Regions that survived a false discovery rate of q<0.05 are shown in the brain map. (B) Same as panel A but simulated from pMFM using the best model parameters from the validation set and structural connectivity from the test set. Regions that survived a false discovery rate of q<0.05 are shown in the brain map. Source data are provided as a Source Data file.

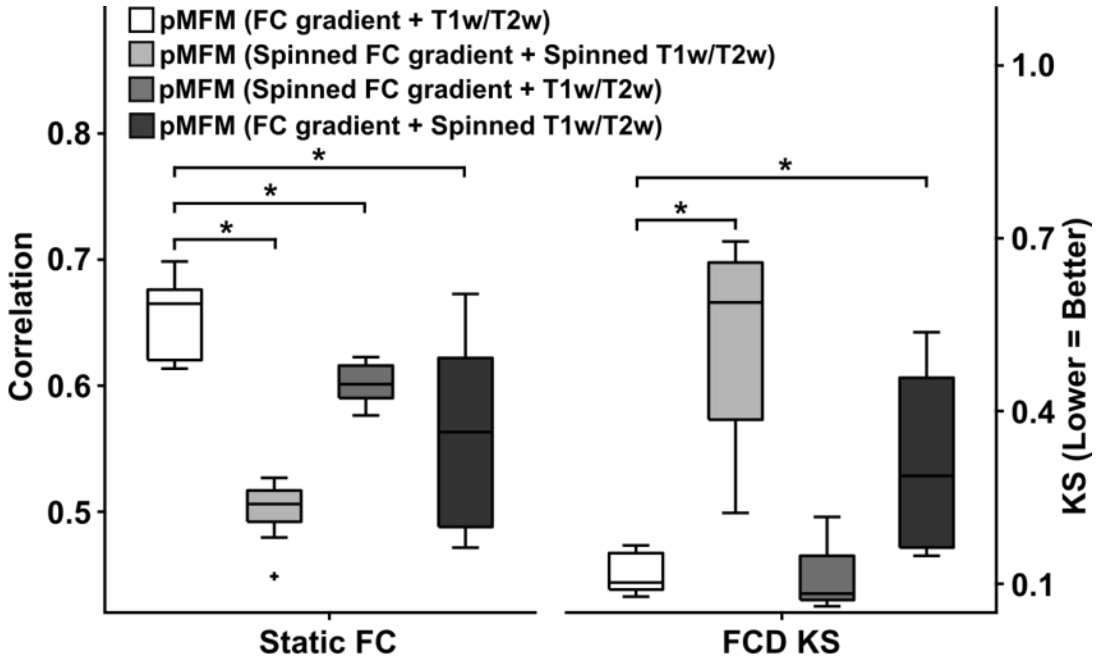


Figure S9. Parameterization with FC gradient and T1w/T2w map led to more realistic static FC and FCD compared with randomly rotated versions of T1w/T2w map and/or FC gradient in the test set. To generate the rotated versions of T1w/T2w map (or FC gradient), we performed 1000 random rotations and picked the rotated map with the smallest absolute correlation with the original T1w/T2w map (or FC gradient). The MFMs with rotated parameterizations were optimized using the same training-validation-test procedure in Figure 1B. Each boxplot comprises 10 correlation values (left) or 10 KS statistic (right) based on the 10 best candidate sets from the validation set. The boxes show the inter-quartile range (IQR) and the median. Whiskers indicate 1.5 IQR. Black crosses represent outliers. * indicates statistical significance after multiple comparisons correction with false discovery rate (FDR) $q < 0.05$. Source data are provided as a Source Data file.

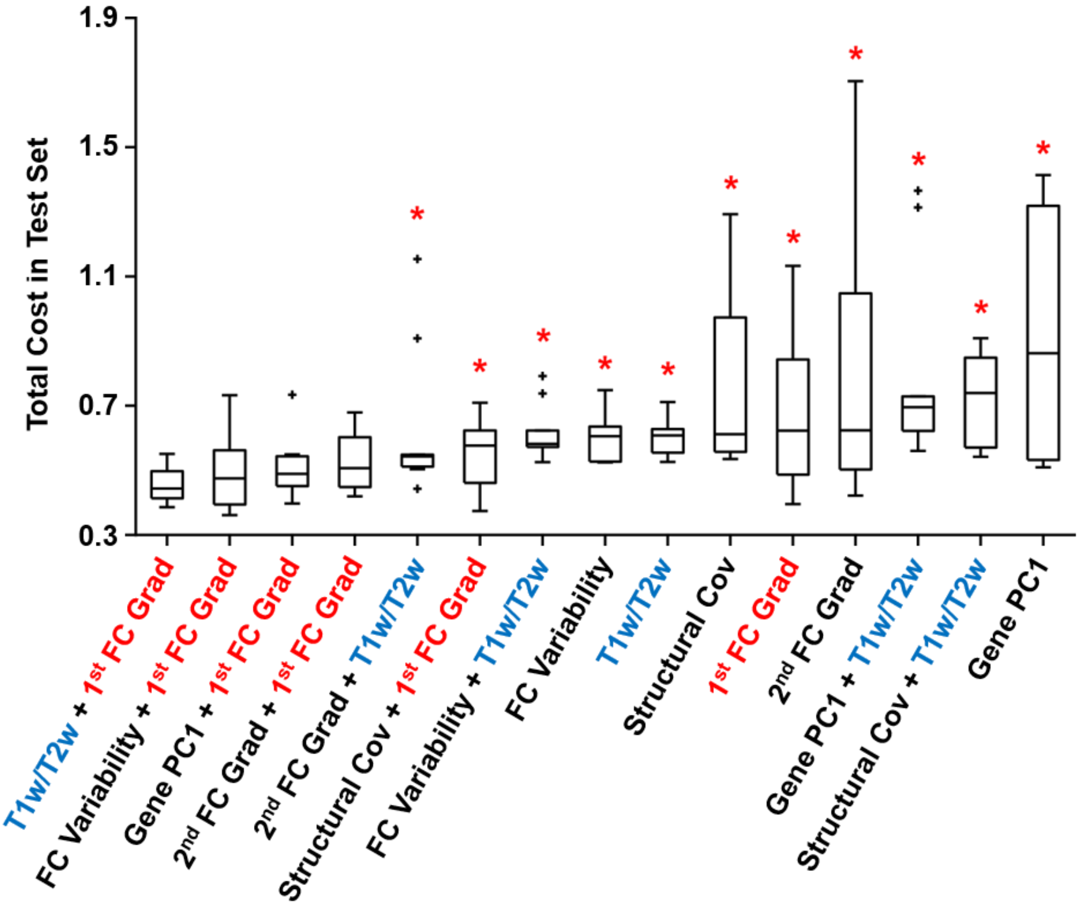


Figure S10. In addition to T1w/T2w map and first FC gradient, we considered other alternative gradient maps, including second FC gradient[7], inter-subject functional connectivity variability map[8], first structural covariance gradient[9] and the first genetic principal component. All parameterizations were optimized using the same training-validation-test procedure in Figure 1B. Each boxplot comprises 10 correlation values (left) or 10 KS statistic (right) based on the 10 best candidate sets from the validation set. The boxes show the inter-quartile range (IQR) and the median. Whiskers indicate 1.5 IQR. Black crosses represent outliers. * indicates that parametrization with T1w/T2w map and first FC gradient was statistically better after multiple comparisons correction with a false discovery rate of $q < 0.05$. In general, inclusion of the first FC gradient was important for performance in the test set. Although combining T1w/T2w map with the first FC gradient led to numerically the best performance in the test set, the improvement was not significant when the T1w/T2w map was replaced with inter-subject FC variability, first genetic principal component or second FC gradient. Source data are provided as a Source Data file.

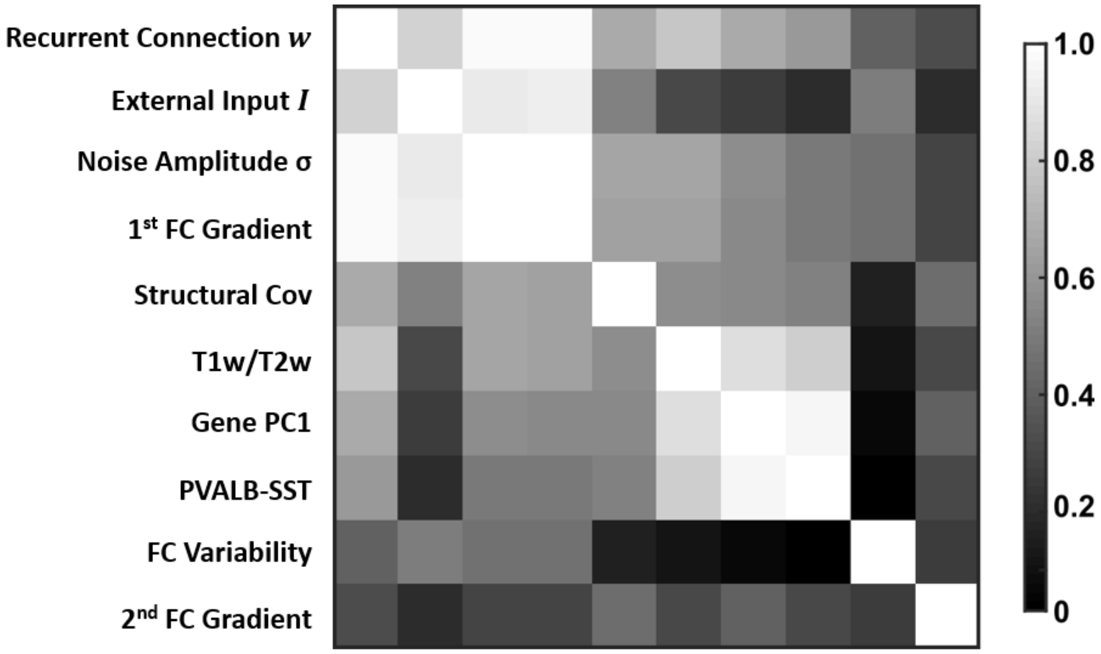


Figure S11. Correlations among the top estimated model parameters ($w$, $I$, $\sigma$) from the original pMFM (Figure 4), first FC gradient, first structural covariance gradient[9], T1w/T2w map, first genetic principal component, PVALB-SST, inter-subject functional connectivity variability map[8], and second FC gradient[7]. The first FC gradient was strongly correlated with the estimated model parameters ($w$, $I$, $\sigma$), while the T1w/T2w map was strongly correlated with the first genetic principal component consistent with previous studies[1]. Source data are provided as a Source Data file.

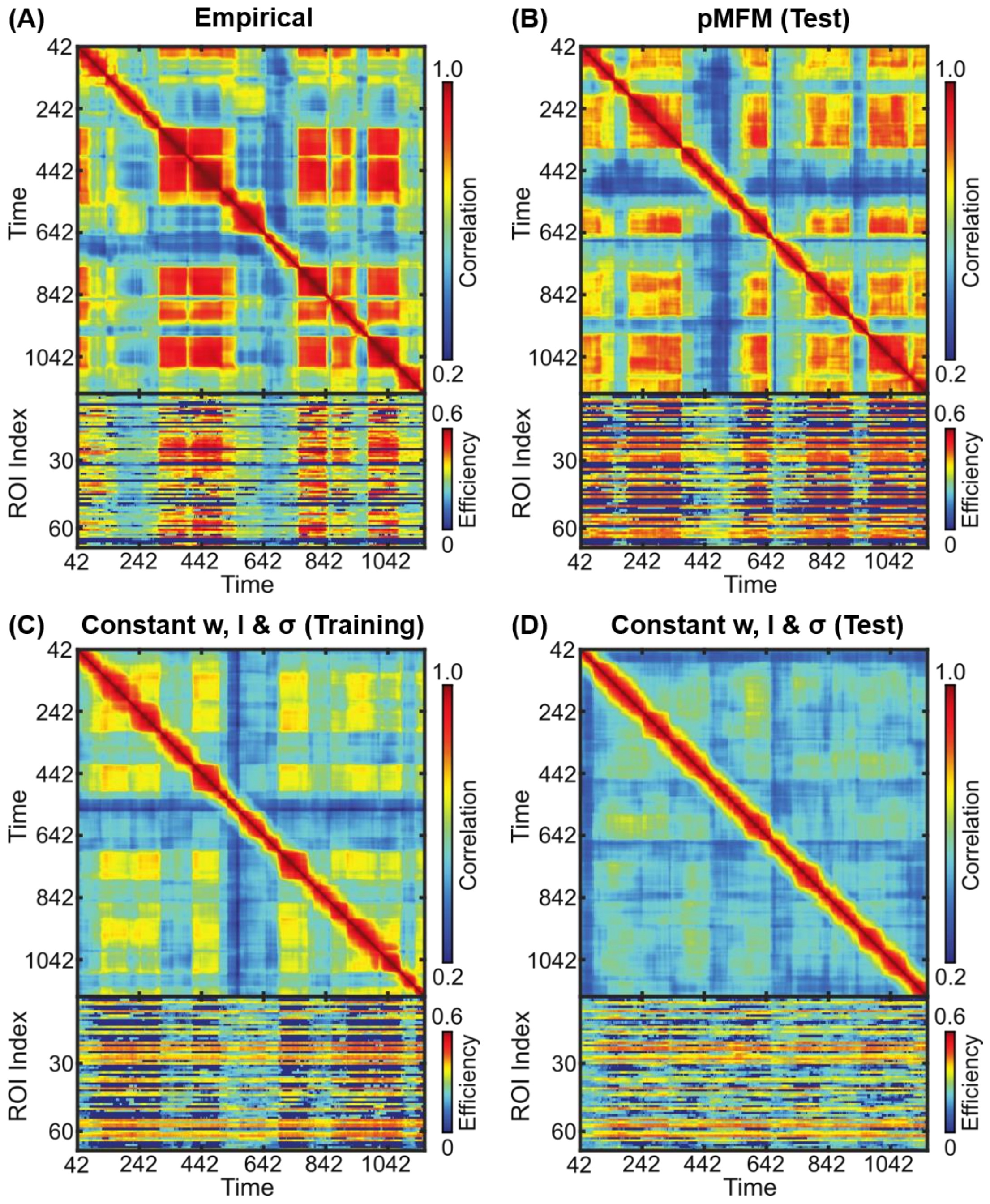


Figure S12. High and low efficiency states in time-varying FC. (A) Empirical FCD from a participant from the HCP test set. The high and low coherent states in the current study corresponded to periods of high and low efficiency[10]. (B) Spatially heterogeneous pMFM was able to generate both high and low efficiency states in the test set. (C) Spatially homogeneous pMFM was able to generate high and low efficiency states in the training set. (D) Spatially homogeneous pMFM was not able to generate high and low efficiency states in the test set. Source data are provided as a Source Data file.

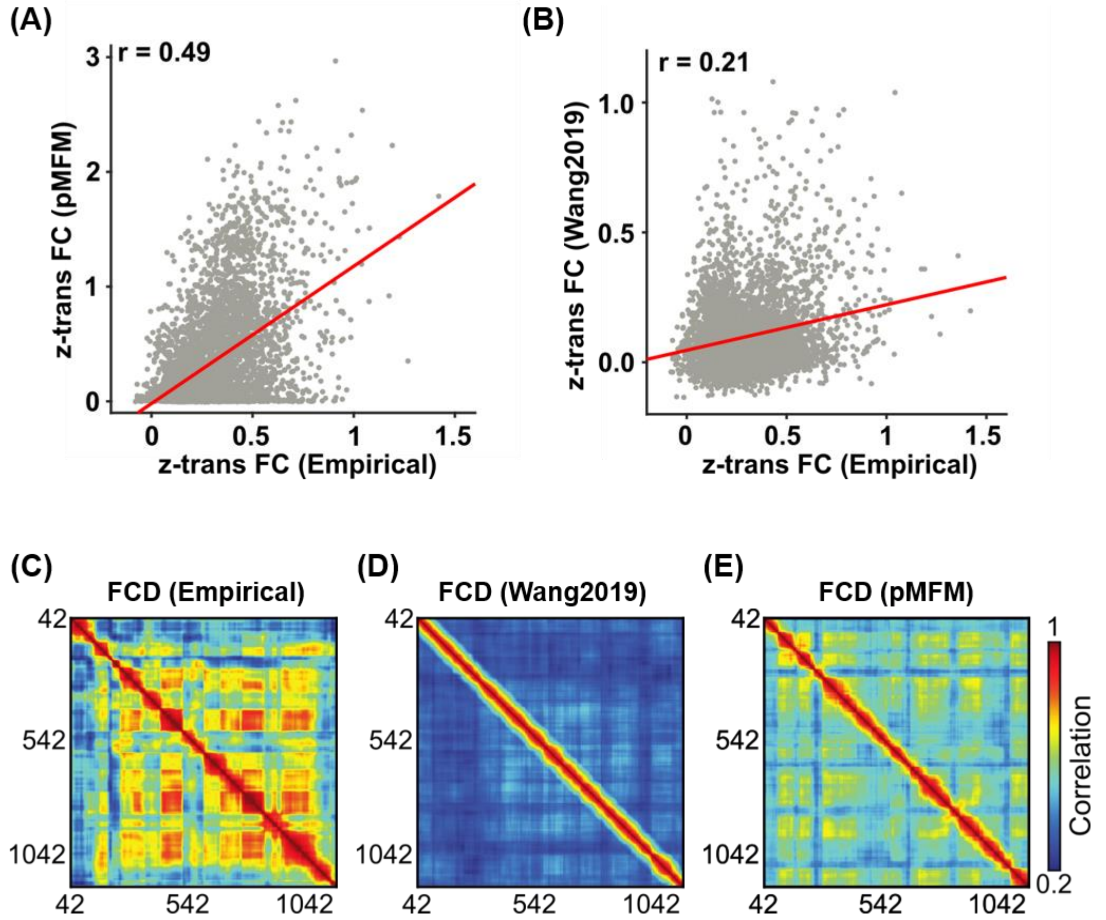


Figure S13. Parametric mean field model (pMFM) generates more realistic static functional connectivity (FC) and functional connectivity dynamics (FCD) than the previous spatially heterogeneous MFM[11] in the 100-region Schaefer parcellation. This figure is similar to Figure 2 but utilizes the 100-region Schaefer parcellation. (A) Agreement (Pearson's correlation) between empirically observed and pMFM-simulated static FC. (B) Agreement (Pearson's correlation) between empirically observed and simulated static FC from Wang 2019. (C) Empirical FCD from a participant from the HCP test set. (D) Simulated FCD from the pMFM using the best model parameters from the validation set using structural connectivity (SC) from the test set. (E) Simulated FCD generated by the previous spatially heterogeneous MFM[11]. Source data are provided as a Source Data file.

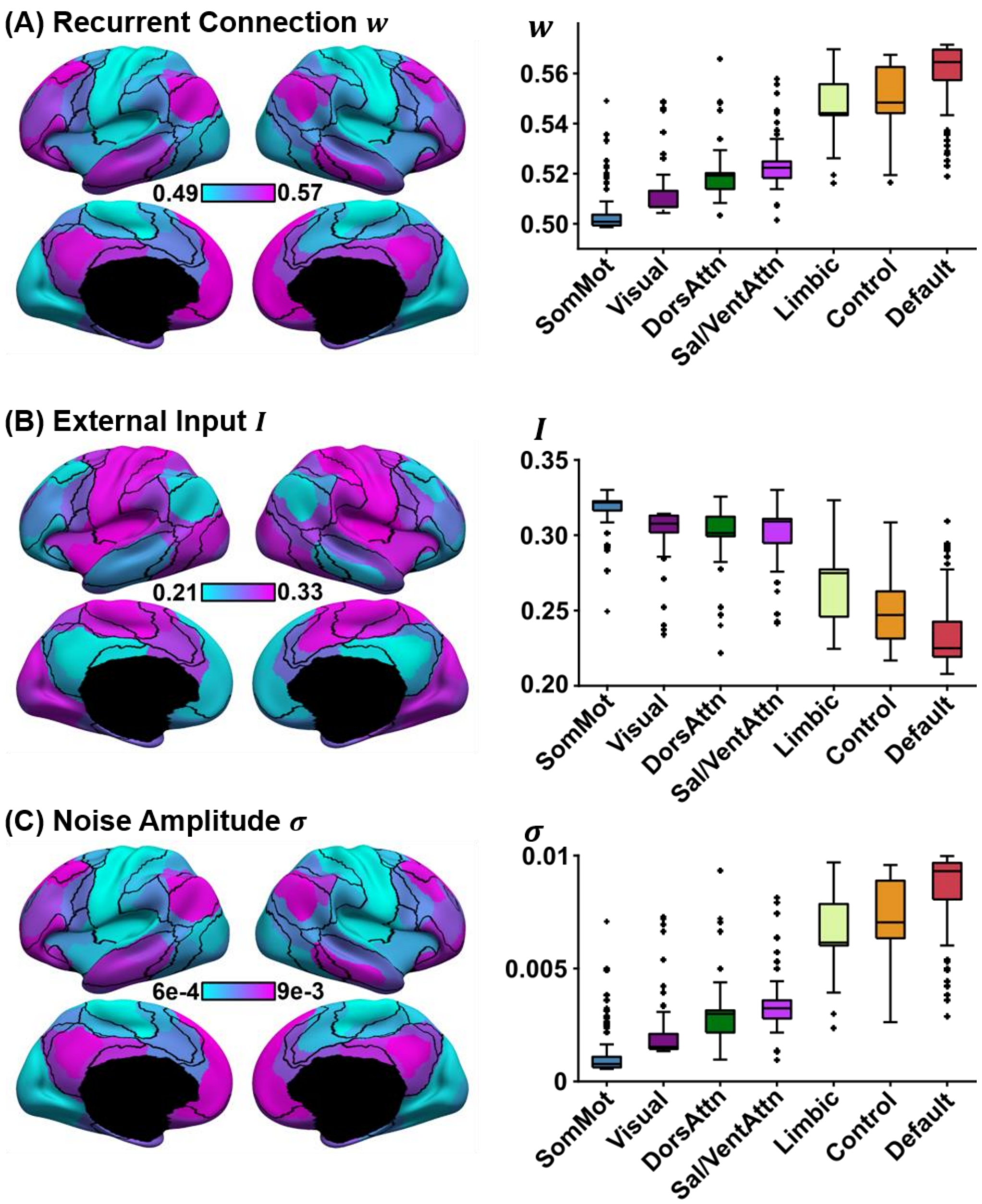


Figure S14. Spatial distribution of recurrent connection strength $w$, external input current $I$, and noise amplitude $\sigma$, and their relationships with resting-state networks in the 100-region Schaefer parcellation. This figure is similar to Figure 4 but utilizes the 100-region Schaefer parcellation. (A) Strength of recurrent connection $w$ in 100 Schaefer cortical ROIs (left) and seven resting-state networks (right). (B) Strength of external input $I$ in 100 Schaefer cortical ROIs (left) and seven resting-state networks (right). (C) Strength of noise amplitude $\sigma$ in 100 Schaefer cortical ROIs (left) and seven resting-state networks (right). The boxplots comprised values obtained by "transferring" the parameter estimates from the 100 Schaefer parcels to all vertices (from the underlying cortical meshes) comprising each anatomical parcel. The vertex wise parameter values were then segregated based on the seven resting-state networks. Therefore, there were 3203, 2478, 1523, 1520, 1067, 1438 and 2886 values comprising the boxplots for somatomotor, visual, dorsal attention, ventral attention,

limbic, control and default networks respectively. The boxes show the inter-quartile range (IQR) and the median. Whiskers indicate 1.5 IQR. Black crosses represent outliers. Recurrent connection strength and noise amplitude increased from sensory-motor to association (limbic, control and default) networks. On the other hand, external input current was the highest in sensory-motor networks and decreased towards the default network. Source data are provided as a Source Data file.

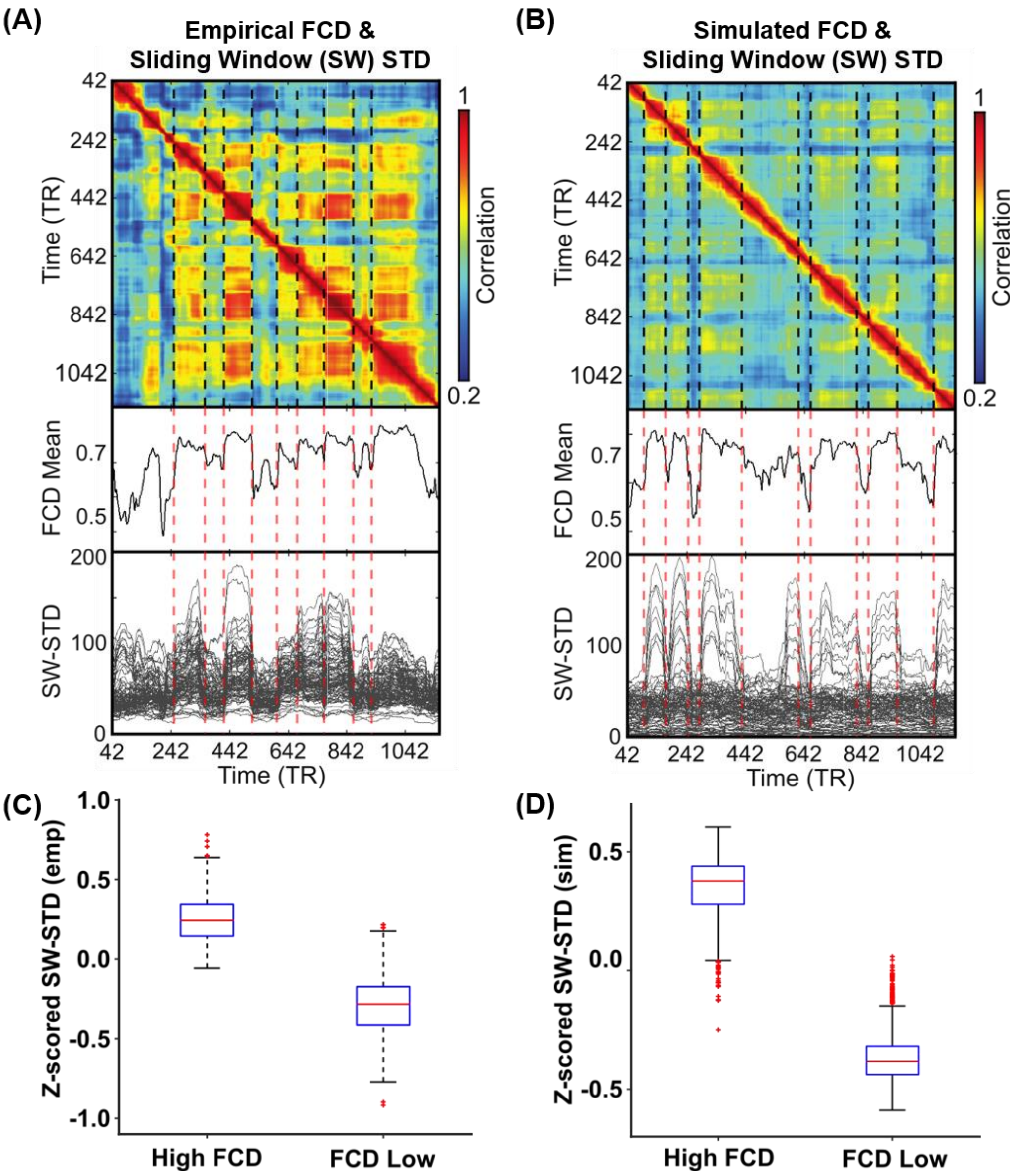


Figure S15. Correspondence between functional connectivity dynamics (FCD) and time-varying amplitude of regional fMRI time courses using the 100-region Schaefer parcellation. This figure is similar to Figure 5 but utilizes the 100-region Schaefer parcellation. (A) Top panel shows empirical FCD matrix of a HCP test participant. The middle panel shows the FCD mean time course obtained by averaging the rows of the FCD matrix from the top panel. The bottom panel shows the standard deviation of each regional fMRI time course within each sliding window (SW-STD). The color of the lines corresponds to the correlation between the first derivative of the FCD mean time course and the first derivative of the SW-STD time courses. Sharp transitions in SW-STD corresponded to sharp FCD transitions (red dashed lines). (B) Same as panel A, but simulated from pMFM using the best model parameters from the validation set and structural connectivity from the test set. (C) SW-STD during coherent (high FCD mean) and incoherent (low FCD mean) states. Boxplots illustrate variation across HCP test participants. Coherent states were characterized by large amplitude (STD) in fMRI signals (p = 4.4e-115). The p value was computed from a two-sided t-test and

survived the false discovery rate ($q < 0.05$). (D) Same as panel C but simulated from pMFM. There are 349 and 1000 independent samples for the boxplots in (C) and (D) respectively. The boxes show the inter-quartile range (IQR) and the median. Whiskers indicate 1.5 IQR. Red crosses represent outliers. Source data are provided as a Source Data file.

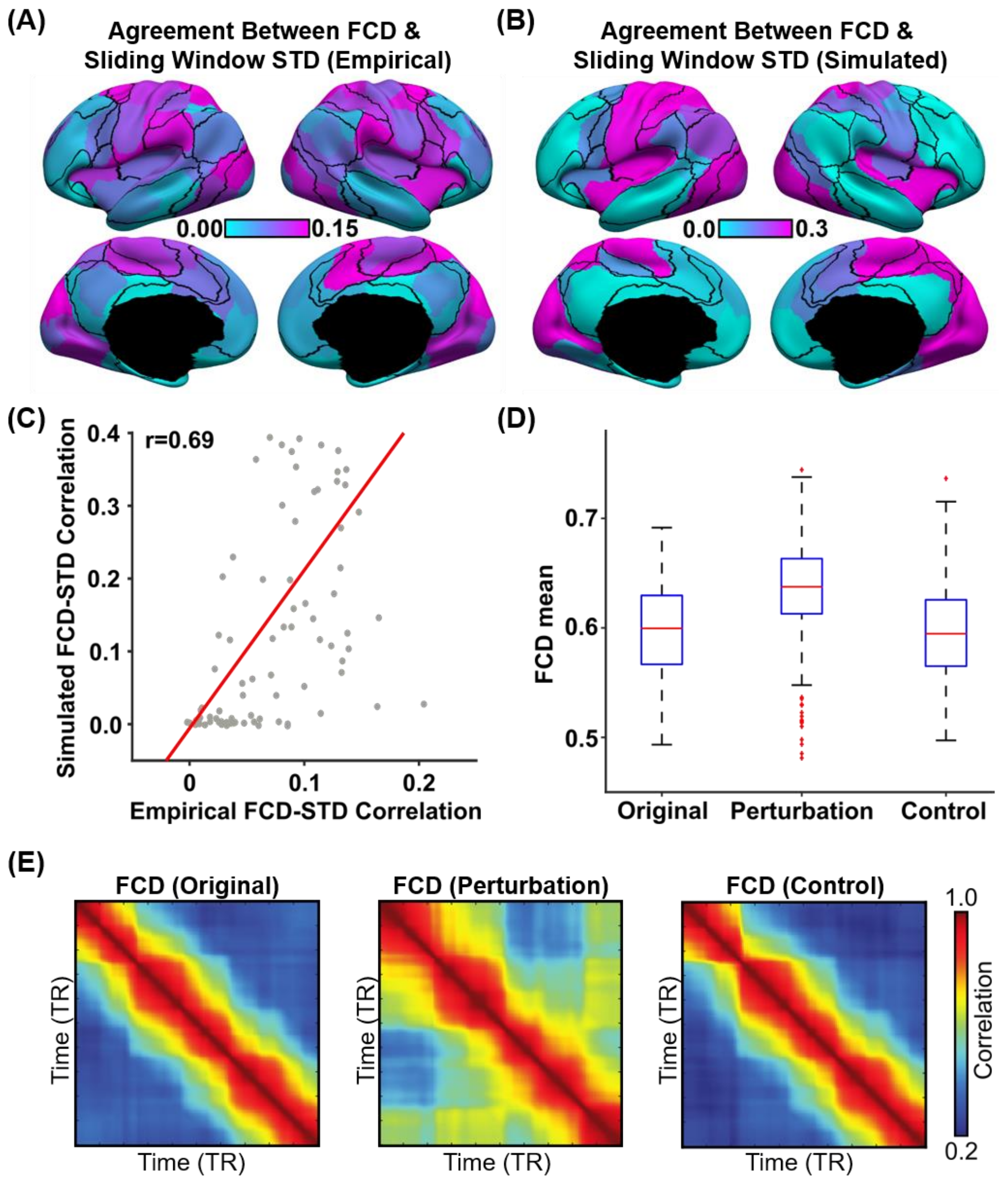


Figure S16. Sensory-motor regions drive sharp transitions in functional connectivity dynamics (FCD) in the 100-region Schaefer parcellation. This figure is similar to Figure 6 but utilizes the 100-region Schaefer parcellation (A) FCD-STD correlations obtained by correlating the first derivative of the FCD mean time course and the first derivative of the SW-STD time course of each cortical region. (B) Same as panel A but simulated from pMFM (C) Correlation between empirical and simulated FCD-STD correlation spatial maps from panels B and C, showing strong correspondence between empirical and simulated results. (D) Casual perturbation of top 5 FCD-STD correlated regions (panel B) during the incoherent state (low FCD mean) led to transition into the coherent state (high FCD mean). As a control analysis, perturbation of the bottom 5 FCD-STD correlated regions (panel B) during the incoherent state (low FCD mean) did not lead to a state change (FCD mean remains low). There are 291 independent samples for each boxplot in (D). The boxes show the inter-quartile range (IQR) and the median. Whiskers indicate 1.5 IQR. Red crosses represent outliers. (E) Example FCD from the perturbation experiments. (Left) original

incoherent state. (Middle) perturbation of top 5 FCD-STD correlated regions (sensory-motor drivers). (Right) perturbation of bottom 5 FCD-STD correlated regions. Source data are provided as a Source Data file.

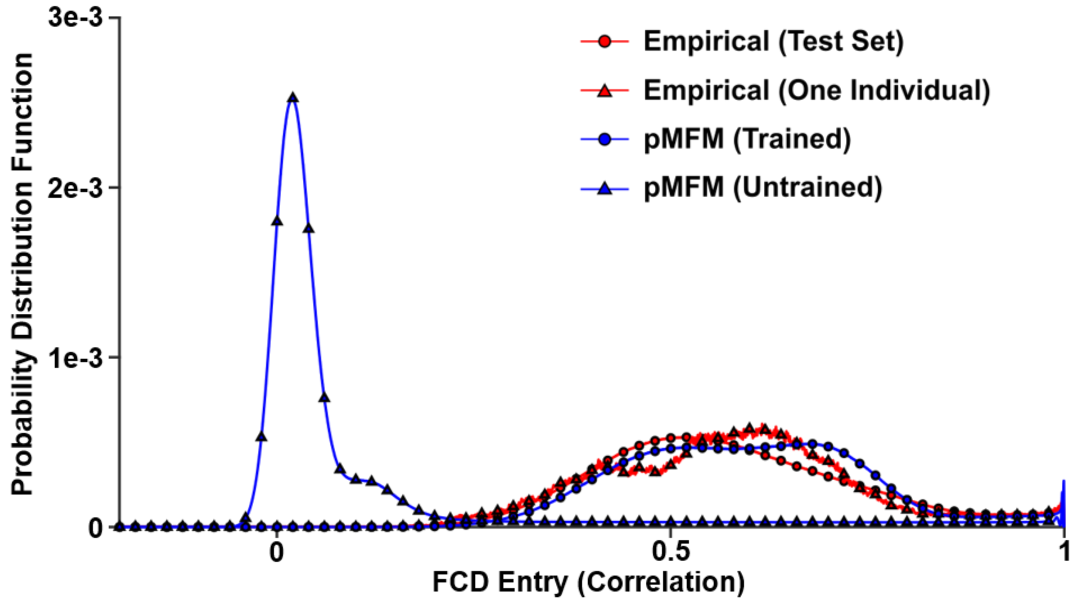


Figure S17. Functional connectivity dynamics (FCD) probability distribution functions (pdfs) of empirical and simulated data. At the individual-level, the FCD pdf was bimodal. At the group-level, the FCD pdf was unimodal. Interestingly, although the pMFM was fitted to the group-level FCD pdf, the resulting FCD distribution still exhibits hints of bimodality. Source data are provided as a Source Data file.

## References


1 Burt, J. B. *et al.* Hierarchy of transcriptomic specialization across human cortex captured by structural neuroimaging topography. *Nat Neurosci* **21**, 1251-1259, doi:10.1038/s41593-018-0195-0 (2018).

2 Anderson, K. M. *et al.* Convergent molecular, cellular, and cortical neuroimaging signatures of major depressive disorder. *Proc Natl Acad Sci U S A* **117**, 25138-25149, doi:10.1073/pnas.2008004117 (2020).

3 Hansen, N. The CMA evolution strategy: a comparing review. *Towards a new evolutionary computation*, 75-102 (2006).

4 Davendra, D. & Zelinka, I. *Self-Organizing Migrating Algorithm: Methodology and Implementation*. Vol. 626 (Springer, 2016).

5 Ilievski, I., Akhtar, T., Feng, J. & Shoemaker, C. in *Proceedings of the AAAI Conference on Artificial Intelligence.*

6 Cocchi, L., Gollo, L. L., Zalesky, A. & Breakspear, M. Criticality in the brain: A synthesis of neurobiology, models and cognition. *Prog Neurobiol* **158**, 132-152, doi:10.1016/j.pneurobio.2017.07.002 (2017).

7 Margulies, D. S. *et al.* Situating the default-mode network along a principal gradient of macroscale cortical organization. *Proc Natl Acad Sci U S A* **113**, 12574-12579, doi:10.1073/pnas.1608282113 (2016).

8 Mueller, S. *et al.* Individual variability in functional connectivity architecture of the human brain. *Neuron* **77**, 586-595, doi:10.1016/j.neuron.2012.12.028 (2013).

9 Valk, S. L. *et al.* Shaping brain structure: Genetic and phylogenetic axes of macroscale organization of cortical thickness. *Sci Adv* **6**, eabb3417, doi:10.1126/sciadv.abb3417 (2020).

10 Zalesky, A., Fornito, A., Cocchi, L., Gollo, L. L. & Breakspear, M. Time-resolved resting-state brain networks. *Proc Natl Acad Sci U S A* **111**, 10341-10346, doi:10.1073/pnas.1400181111 (2014).

11 Wang, P. *et al.* Inversion of a large-scale circuit model reveals a cortical hierarchy in the dynamic resting human brain. *Sci Adv* **5**, eaat7854, doi:10.1126/sciadv.aat7854 (2019).
