# Supplementary material for: Sensory-motor cortices shape functional connectivity dynamics in the human brain
Source: Nat Commun. 2021 Nov 4;12:6373. doi: 10.1038/s41467-021-26704-y (PMC8568904; doi:10.1038/s41467-021-26704-y)
Supplement: Supplementary file 3 — Reporting Summary [file 41467_2021_26704_MOESM3_ESM.pdf]

nature portfolio

Corresponding author(s): B.T. Thomas Yeo

Last updated by author(s): Aug 24, 2021

Reporting Summary

Nature Portfolio wishes to improve the reproducibility of the work that we publish. This form provides structure for consistency and transparency in reporting. For further information on Nature Portfolio policies, see our Editorial Policies and the Editorial Policy Checklist.

Statistics

For all statistical analyses, confirm that the following items are present in the figure legend, table legend, main text, or Methods section.

n/a	Confirmed	
☐	☒	The exact sample size (n) for each experimental group/condition, given as a discrete number and unit of measurement
☐	☒	A statement on whether measurements were taken from distinct samples or whether the same sample was measured repeatedly
☐	☒	The statistical test(s) used AND whether they are one- or two-sided<br>*Only common tests should be described solely by name; describe more complex techniques in the Methods section.*
☐	☒	A description of all covariates tested
☐	☒	A description of any assumptions or corrections, such as tests of normality and adjustment for multiple comparisons
☐	☒	A full description of the statistical parameters including central tendency (e.g. means) or other basic estimates (e.g. regression coefficient) AND variation (e.g. standard deviation) or associated estimates of uncertainty (e.g. confidence intervals)
☐	☒	For null hypothesis testing, the test statistic (e.g. F, t, r) with confidence intervals, effect sizes, degrees of freedom and P value noted<br>*Give P values as exact values whenever suitable.*
☒	☐	For Bayesian analysis, information on the choice of priors and Markov chain Monte Carlo settings
☒	☐	For hierarchical and complex designs, identification of the appropriate level for tests and full reporting of outcomes
☐	☒	Estimates of effect sizes (e.g. Cohen's d, Pearson's r), indicating how they were calculated

Our web collection on statistics for biologists contains articles on many of the points above.

Software and code

Policy information about availability of computer code

Data collection	All data used in the present study came from the WU-Minn HCP Consortium S1200 Release. They are publicly available, accessible at https://www.humanconnectome.org
Data analysis	MATLAB (2018b); Python (3.6); Pytorch (1.0.1); The code is publicly available on Zenodo database. https://doi.org/10.5281/zenodo.5518257

For manuscripts utilizing custom algorithms or software that are central to the research but not yet described in published literature, software must be made available to editors and reviewers. We strongly encourage code deposition in a community repository (e.g. GitHub). See the Nature Portfolio guidelines for submitting code & software for further information.

Data

Policy information about availability of data

All manuscripts must include a data availability statement. This statement should provide the following information, where applicable:

- Accession codes, unique identifiers, or web links for publicly available datasets
- A description of any restrictions on data availability
- For clinical datasets or third party data, please ensure that the statement adheres to our policy

The Human Connectome Project (HCP) data are publicly available: https://www.humanconnectome.org/study/hcp-young-adult/document/1200-subjects-data-release

Field-specific reporting

Please select the one below that is the best fit for your research. If you are not sure, read the appropriate sections before making your selection.

[x] Life sciences   [ ] Behavioural & social sciences   [ ] Ecological, evolutionary & environmental sciences

For a reference copy of the document with all sections, see nature.com/documents/nr-reporting-summary-flat.pdf

Life sciences study design

All studies must disclose on these points even when the disclosure is negative.

Sample size	The sample size was determined by the HCP S1200 release, which is 1113 subjects.
Data exclusions	All subjects were included if they had completed brain scans of rsfMRI and dMRI in HCP S1200 release. We excluded 61 subjects because they do not have completed rsfMRI or dMRI.
Replication	The main results were replicated on the same dataset with 1052 subjects using a different resolution functional parcellation. All attempts at replication were successful.
Randomization	The 1052 subjects were randomly divided into training (N=351), validation (N=350) and test (N=351) sets.
Blinding	Blinding is not relevant to this study as no data collection was involved

Reporting for specific materials, systems and methods

We require information from authors about some types of materials, experimental systems and methods used in many studies. Here, indicate whether each material, system or method listed is relevant to your study. If you are not sure if a list item applies to your research, read the appropriate section before selecting a response.

Materials & experimental systems

n/a	Involved in the study	
[x]	[ ]	Antibodies
[x]	[ ]	Eukaryotic cell lines
[x]	[ ]	Palaeontology and archaeology
[x]	[ ]	Animals and other organisms
[ ]	[x]	Human research participants
[x]	[ ]	Clinical data
[x]	[ ]	Dual use research of concern

Methods

n/a	Involved in the study	
[x]	[ ]	ChIP-seq
[x]	[ ]	Flow cytometry
[ ]	[x]	MRI-based neuroimaging

Human research participants

Policy information about studies involving human research participants

Population characteristics	See above
Recruitment	1113 young adult (ages 22-35) participants were recruited from families with twins and non-twin siblings in Human Connectome Project (HCP). However, our lab was not personally involved in the recruitment. More information can be found at https://www.humanconnectome.org/study/hcp-young-adult/project-protocol/recruitment.
Ethics oversight	Although the data was not collected by us, our study is approved by the National University of Singapore Institutional Review Board (IRB).

Note that full information on the approval of the study protocol must also be provided in the manuscript.

Magnetic resonance imaging

Experimental design

Design type	HCP provided both resting-state fMRI and task fMRI. However, we only consider resting-state fMRI in our study
Design specifications	Each participant underwent 4 runs of resting-state and each rs-fMRI run was acquired with a repetition time (TR) of 0.72s at 2mm isotropic resolution and lasted for 14.4 min.

Behavioral performance measures: We did not consider any behavioural measure in our study.

Acquisition

Imaging type(s): functional & diffusion

Field strength: 3T

Sequence & imaging parameters: All participants were scanned on a customized Siemens 3T Skyra using a multi-band sequence. Four resting-state fMRI (rs-fMRI) runs were collected for each participants in two sessions on two different days. Each rs-fMRI run was acquired with a repetition time (TR) of 0.72s at 2mm isotropic resolution and lasted for 14.4 min.

Area of acquisition: Whole brain scan

Diffusion MRI ☒ Used ☐ Not used

Parameters: The diffusion imaging consisted of 6 runs, each lasting approximately 9 minutes and 50 seconds. Diffusion weighting consisted of 3 shells of b = 1000, 2000, and 3000 s/mm2 with an 555 approximately equal number of weighting directions on each shell.

Preprocessing

Preprocessing software: FSL 5.0.6; FreeSurfer 5.3.0; Details of the HCP preprocessing can be found in the HCP S1200 manual

Normalization: Non-linear surface registration. We used the MSM registration provided by HCP. See HCP S1200 manual for more details.

Normalization template: Conte69 registered standard mesh with ~32k vertices (see HCP S1200 manual)

Noise and artifact removal: We utilized rs-fMRI data, which had already been projected to fsLR surface space, denoised with ICA-FIX and smoothed by 2mm

Volume censoring: Voxels with a temporal coefficient of variance greater than 0.5 standard deviations of their local neighborhood (sigma=5mm) are excluded from volume to surface mapping (see HCP S1200 manual)

Statistical modeling & inference

Model type and settings: Generative Model; Biophysical Model; Neural Mass Model

Effect(s) tested: We tested whether we could simulated realistic fMRI signal based on the diffusion MRI data by utilizing the dynamic mean field model.

Specify type of analysis: ☒ Whole brain ☐ ROI-based ☐ Both

Statistic type for inference (See Eklund et al. 2016): We use functional connectivity measures in our study, so we were not interested in clusters

Correction: False Discovery Rate

Models & analysis

n/a	Involved in the study
☐	☒ Functional and/or effective connectivity
☒	☐ Graph analysis
☐	☒ Multivariate modeling or predictive analysis

Functional and/or effective connectivity: Pearson's correlation

Multivariate modeling and predictive analysis: We utilized dynamic mean field model to generate simulated fMRI signals and evaluate the performance of the estimated model parameters based on two metrics from static and dynamic perspective: Pearson correlation between empirical and simulated functional connectivity and Kolmogorov–Smirnov statistics between empirical and simulated functional connectivity dynamics.
